# Impact of Sandblasting on Morphology, Structure and Conductivity of Zirconia Dental Ceramics Material

**DOI:** 10.3390/ma14112834

**Published:** 2021-05-25

**Authors:** Marko Jakovac, Teodoro Klaser, Borna Radatović, Arijeta Bafti, Željko Skoko, Luka Pavić, Mark Žic

**Affiliations:** 1 Department of Prosthodontics, School of Dental Medicine, University Zagreb, Gundulićeva 5, 10000 Zagreb, Croatia; jakovac@sfzg.hr; 2 Department of Physics, Faculty of Science, University of Zagreb, Bijenička, c. 32, 10000 Zagreb, Croatia; tklaser@phy.hr (T.K.); zskoko@phy.hr (Ž.S.); 3 Center for Excellence for Advanced Materials and Sensing Devices, Institute of Physics, 10000 Zagreb, Croatia; bradatovic@ifs.hr; 4 Faculty of Chemical Engineering and Technology, University of Zagreb, Marulićev trg 19, 10000 Zagreb, Croatia; abafti@fkit.hr; 5 Ruđer Bošković Institute, P.O. Box 180, 10000 Zagreb, Croatia; lpavic@irb.hr

**Keywords:** zirconia, sandblasting, PXRD, impedance, equivalent electrical circuit (EEC), conductivity, dental material

## Abstract

Over the last decade, zirconia (ZrO_2_)-based ceramic materials have become more applicable to modern dental medicine due to the sustained development of diverse computer-aided design/computer-aided manufacturing (CAD/CAM) systems. However, before the cementation and clinical application, the freshly prepared zirconia material (e.g., crowns) has to be processed by sandblasting in the dental laboratory. In this work, the impact of the sandblasting on the zirconia is monitored as changes in morphology (i.e., grains and cracks), and the presence of impurities might result in a poor adhesive bonding with cement. The sandblasting is conducted by using Al_2_O_3_ powder (25, 50, 110 and 125 µm) under various amounts of air-abrasion pressure (0.1, 0.2, 0.4 and 0.6 MPa). There has been much interest in both the determination of the impact of the sandblasting on the zirconia phase transformations and conductivity. Morphology changes are observed by using Scanning Electron Microscope (SEM), the conductivity is measured by Impedance Spectroscopy (IS), and the phase transformation is observed by using Powder X-Ray Diffraction (PXRD). The results imply that even the application of the lowest amount of air-abrasion pressure and the smallest Al_2_O_3_ powder size yields a morphology change, a phase transformation and a material contamination.

## 1. Introduction

A great number of scientific studies today are based on clinical research in the field of implantology and disorders in the oral cavity [1,2,3]. However, current computer-aided design/computer-aided manufacturing (CAD/CAM) technology exhibits an accelerated development due to a tenable progress in dental materials development [4,5,6,7,8,9]. One of the most utilized ceramic biomaterials that possesses a unique quality and covers a variety of dental indications is based on zirconia (ZrO_2_) [10,11,12]. These dental materials possess both great toughness and strength, although there might be some issues related to chipping [13,14,15]. ZrO_2_-based materials are also used in different fields of science and technology, such as solid electrolytes in solid oxide fuel cells (SOFC) [16,17,18].

Due to the wide applicability of ZrO_2_-based materials, they are engineered to have distinct properties [10,11,17]. For example, when zirconia is doped by Y^3+^, it becomes stable in the tetragonal phase at room temperature [10]. This type of zirconia-based material is named yttria stabilized-tetragonal zirconia polycrystals (Y-TZP) and is commonly used as a structural ceramic [19,20]. Y-TZP is especially suitable for restorative dentistry due to its chemical and mechanical stability as well as high mechanical strength [9]. However, a tetragonal phase can be transformed into a monoclinic phase which is accompanied by an increase in volume (and by crack absorption) [21]. Such a transformation can induce a severe clinical implication such as increased risk of fracture. The structural properties of this type of material can be studied by Powder X-ray Diffraction (PXRD) [22,23,24]. By varying the amount of Y^3+^, it is possible to finely tune Y-TZP properties such as translucence, toughness and hardness that are especially important for dental indications and restorations. 

On the other hand, the amount of Y^3+^ also governs the ratio of oxygen vacancies and, consequently, the ionic oxygen–ion conductivity [16]. Nonetheless, Y-TZP conductivity is also controlled by intragranular and intergranular resistivities [17,18], structural properties [17] and impurities [25]. The contribution of both ohmic and ionic conductivity to total conductivity is especially relevant for solid zirconia-based electrolytes utilized in SOFC technology [18]. It should be noted that the Impedance Spectroscopy (IS) technique is extremely capable of distinguishing between intragranular and intergranular conductivity [17], which facilitates the conductivity study of ZrO_2_-based materials [18,25].

Before the dental application, Y-TZP material has to be processed for different indications by CAD/CAM technology which is nowadays a widely accepted strategy [26,27,28]. Afterwards, air-abrasion, i.e., sandblasting, is utilized to ensure a strong and durable bonding between Y-TZP and self-adhesive resin cements [29]. However, CAD/CAM treatment can also induce some undesirable microscopic damages to the Y-TZP surface such as voids that can act as a center for aging. Until now, it has not been confirmed whether these voids can be sealed by sandblasting, although its impact on zirconia-based materials has indeed been thoroughly studied [15,20,30,31,32,33,34]. Moreover, Y-TZP material has to be sintered, a task that can introduce a small number of impurities [35]. Thus, sandblasting with Al_2_O_3_ before the application is still a common approach [33] for several reasons: first, to remove impurities and contaminants [35]; second, to obtain certain properties such as increased surface and wettability [27,28]; third, to improve adhesion during cementation [20,29,36]. 

The majority of studies that investigated the sandblasting effect have been conducted in strictly controlled conditions [20,27,28,37,38]. However, these conditions are usually unavailable in ordinary dental laboratories. As sintering is mostly conducted in dental laboratories, there is also a high risk of materials contamination [35], which can induce changes in the electrical conductivity [25]. Nonetheless, the surface contaminants generally affect adhesion to Y-TZP material [39,40]; thus, a portion of contaminants should be monitored. Intriguingly, electrical measurements have not been applied yet to monitor the impact of sandblasting on the amount of impurities in the dental Y-TZP material. If the aforementioned short analysis is taken into consideration, the impact of the sandblasting in a dental laboratory on the surface, structural and conductivity properties of Y-TZP has not been thoroughly addressed yet.

This research was mainly devoted to studying the effect of sandblasting on Y-TZP material conducted in an ordinary dental laboratory. The special interest was focused on determining the impact of the Al_2_O_3_ powder size and pressure on the surface, structure and conductivity properties.

Overall, the main highlights of this study are understanding that the sandblasting can seal up the holes in Y-TZP material and a realization that the electrical measurements can be used to monitor a portion of impurities in the material.

## 2. Materials and Methods

### 2.1. Preparation of Samples

In this work, IPS e.max^®^ ZirCAD Prime All-Ceramics Y-TZP system (Ivoclar Vivadent, Shaan, Liechtenstein) was studied. From the above Y-TZP system (i.e., material), a total of 17 sample disks (1 mm thickness and 10 mm diameter) were produced in the dental laboratory by using a dental milling unit (Cerec, MCX 5, Dentsply Sirona, Bensheim, Germany). The disk dimensions were carefully chosen so that the sample disks could be analysed by all experimental techniques without any additional adjustment in size. According to the manufacturer’s recommendations, they were additionally sintered (inLab Profire, Dentsply Sirona, Bensheim, Germany). 1 sample disk was left as–prepared (the control ZR sample), while 16 other sample disks were sandblasted by different Al_2_O_3_ (Renfert GmbH, Hilzingen, Germany) powders (25, 50, 110 and 125 µm) at 0.1, 0.2, 0.4 and 0.6 MPa. The prepared sample disks’ names and the sandblasting conditions are given in Table 1.

The sandblasting procedure applied herein was similar to the one that can be found in the literature (e.g., [22,27,28]). To be exact, the blasting nozzle was placed perpendicularly to the sample surfaces at a distance of 10 mm. The air-abrasion was performed vertically on the sample. However, in contrast to references [22,27,28], the sandblasting in this study was intentionally conducted manually, so that the treatment of the samples resembled that performed at a dental technician’s practice. The blasting of the sample’s disk surface was conducted for 60 s (both sides were sandblasted). However, finding the optimal conditions for the sandblasting of Y-TZP based materials is not a straightforward task [22].

### 2.2. Surface Investigation Methods

The morphology of the control and sandblasted samples was investigated by tungsten filament SEM VEGA 3 manufactured by TESCAN Ltd. (Saint Petersburg, Russia). The specimens were not covered by a conductive material. The image acquisition was done in resolution mode with a secondary electron (SE) detector at 10 mm working distance and 5 kV acceleration voltage. For Energy Dispersive X-ray Spectrometry (EDX), Bruker’s XFlash 6l30 detector (Brno, Czech Republic) was used, whereas ESPRIT 2.1 software (version 2.1) was employed for the analysis of spectra. The spectra were taken at 15 mm working distance and 5 kV acceleration voltage. 

### 2.3. Structural Investigation Methods

Powder X-ray Diffraction (PXRD) measurements were recorded with a Bruker Discover D8 diffractometer (Karlsruhe, Germany), equipped with a LYNXEYE XE-T detector, in Bragg–Brentano geometry (1D) using Cu*K*α radiation (1.54 Å) in the angular range 2*Θ* 10–70° with a step size of 0.02° and measuring time of 27 s/step.

### 2.4. Electrical Properties

SC7620 sputter coater (Quorum Technologies Ltd., Laughton, East Sussex, UK) was used to sputter gold electrodes (7 mm in diameter) onto both sides of the sample disks (~1 mm thick). An impedance analyzer (Novocontrol Alpha-AN dielectric spectrometer, Novocontrol Technologies GmbH & Co. KG, Hundsangen, Germany) was used to collect the complex impedance measurements from 0.01 Hz to 1 MHz. The temperature range was from 303 to 483 K and the temperature accuracy was ±0.5 K.

## 3. Results and Discussion

### 3.1. SEM Investigations

**Figure 1:**
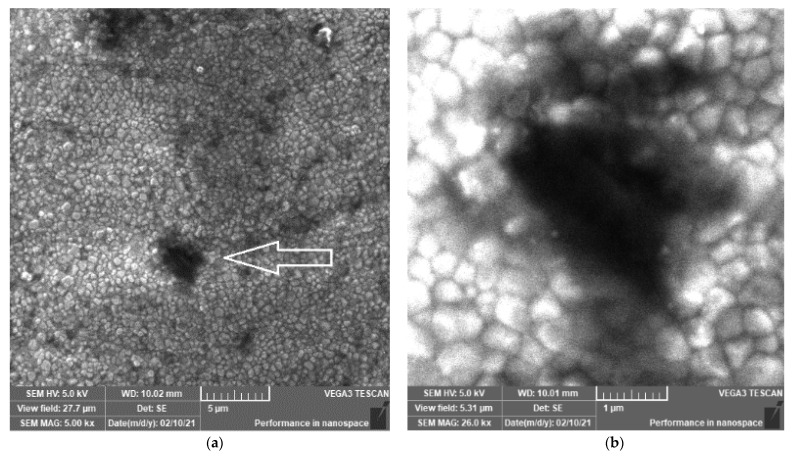
SEM images of different magnifications (×5000 (**a**) and ×25,000 (**b**)) of the ZR control sample prepared by CAD/CAM. The morphology of ZR is characterized by grains (250–500 nm), grain boundaries and several voids (0.5–5 μm in diameter). Symbol reference: white arrow indicates hole/void.

SEM images of the ZR (control) sample disk, which was not sandblasted with Al_2_O_3_, are shown in Figure 1. There are many different surface treatments of ZrO_2_-based dental materials and the sandblasting is the most common one [15,34,41], although there are studies that utilized, e.g., Nd:YAG laser for the same purpose [36,42]. In this study, both images display the expected morphology of the sintered yttria stabilized-tetragonal zirconia polycrystal (Y-TZP) material. Y-TZP materials are commonly used in, e.g., dental medicine for restoration and they were a focus of many different investigations [22,27,43]. The ZR sample is characterized by grain boundaries and grains of different sizes (250–500 nm) and a similar morphology was reported here [27]. Additionally, these grains (Figure 1) should not be too large as they can be transformed into a monoclinic phase at room temperature [43]. 

Next, the ZR sample was also characterized by voids, i.e., holes (0.5–5 μm in diameter), which might be an outcome of the CAD/CAM treatment. The existence of these holes can increase the stress on the material and, consequently, induce unwanted transformations. However, these voids in the non-sandblasted samples were not observed in similar studies [15,27,34,44]. It should be mentioned that defects (i.e., voids) present centers for the material aging that might accelerate the degradation of the indications [26].

**Figure 2:**
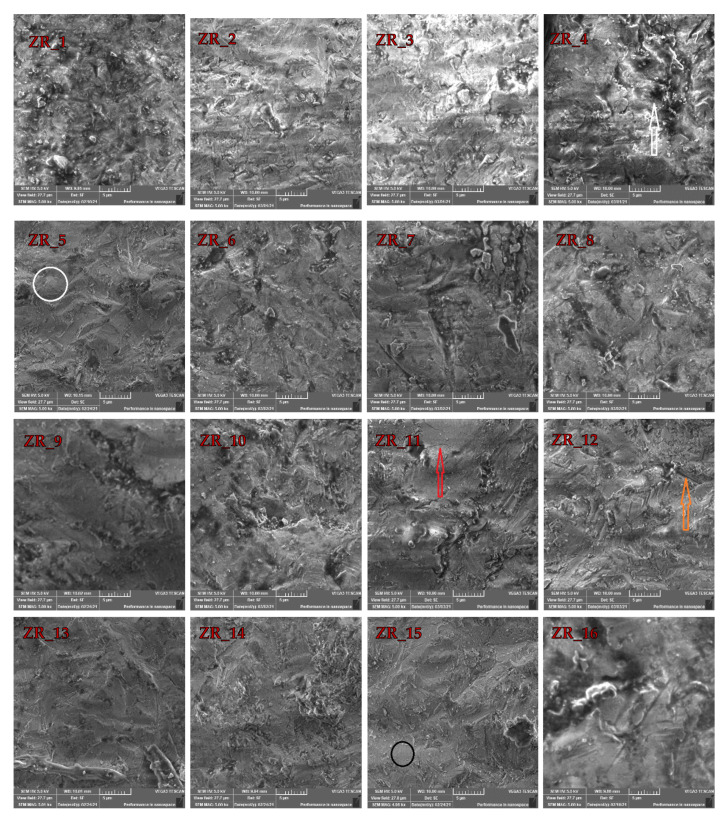
SEM images of the prepared samples (see names in Table 1) obtained after the ZR control sample disk was sandblasted with different Al_2_O_3_ particles at diverse pressures. Symbol reference: white arrow indicates holes, red arrow indicates micro-cracks, orange arrow indicates large cracks, black circle indicates surface melting and white circle indicates plastic deformation.

The sandblasting effect can be observed in Figure 2. The typical Y-TZP morphology (Figure 1) is no longer present as there are no well-defined grain boundaries. According to references [22,27], the kinetic energy of the Al_2_O_3_ particle during the sandblasting is high enough to induce surface melting of the zirconia-based materials. These deformations usually occur due to internal tensile stress and increased temperature induced by the Al_2_O_3_ air-abrasion [27]. Figure 2 also clearly shows that the surfaces of the sandblasted samples (vs. the ZR control sample) are drastically perturbed as the impact of Al_2_O_3_ particles yielded surface damages, such as large cracks (orange arrow), micro-cracks (red arrow), holes (white arrow), surface melting (black circle) and plastic deformations (white circle). However, it appears that the ZR_2 sample (Table 1) shows the lowest portion of surface defects (Figure 2). The presence of these defects is expected as they were also reported in the literature [22,27,28]. These defects also increase roughness which promotes formation of superficial cracks that reduce the strength of restoration [15]. In addition, it was also explained that minor defects obtained by air-abrasion could be “healed” by resin luting agents [45].

The large void (Figure 1a) is not observed in the 16 sandblasted samples displayed in Figure 2. According to the high number of the investigated samples (i.e., 16), it is safe to say that the sandblasting sealed the large void(s) (Figure 1) that can occur due to the milling process. This is an important finding as OH^−^ ions can diffuse via voids (cracks and holes) into zirconia lattice and fill oxygen vacancies, which is a scenario that additionally destabilizes the tetragonal phase [21]. Therefore, the material with the lowest portion of defects on the surface (i.e., ZR_2) is more resistant to aging. Moreover, according to the literature, zirconia-based materials should be sandblasted at low pressures and with alumina < 50 µm [34,46,47]. As the surface damage (which accelerates material aging) of the ZR_2 sample is the lowest, it appears that the sandblasting conditions for this sample are optimal.

### 3.2. EDX Investigations

To extract more data about the impact of the sandblasting, it was necessary to inspect and elaborate the EDX data that are given in Table 2. As the application of EDX in this field of study is a common one [27,28,48], a special focus was applied to examine the atomic fraction (%) of Zr and Al in the sandblasted samples. The presence of Al in the sandblasted samples was expected, especially as it was reported that the traces of Al in the sandblasted dental restoration materials could be detected even after ultrasound cleaning [48]. It appears that the ZR_2 sample (no large holes and micro-cracks) is characterized by the highest proportion of Zr (21.18%). This observation becomes more significant when perceiving that the proportion of Al is the lowest (9.02%) in this sample. The aforementioned statements can be explained by the fact that the size of Al_2_O_3_ and pressure were not high enough to induce surface damages like large holes and micro-cracks, but they were sufficient to induce surface melting and plastic deformations (Figure 2). 

When SEM data are considered, the absence of large holes in the ZR_2 sample can be assigned to the volume ingress that is governed by the structural transformation within Y-TZP [12,21,49]. Therefore, it is fair to conclude that the sandblasting has a positive effect as it can potentially seal (i.e., close) voids obtained during CAM/CAD treatment. Overall, according to SEM and EDX data, the sample ZR_2 has the lowest amount of deformation and Al (i.e., surface impurities); thus, it should be used for dental application. However, to obtain some more general conclusions, one should conduct in vitro analyses, such as artificial aging [26,45]; however, this kind of investigation is currently beyond the scope of this work.

### 3.3. Structural Investigations

**Figure 3:**
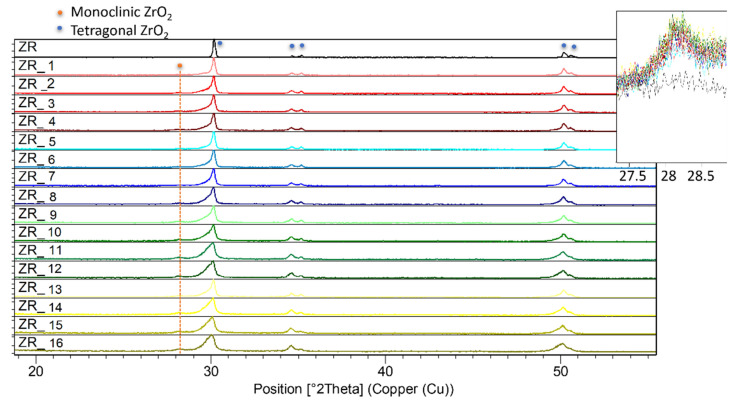
PXRD patterns of the ZR control sample and all the samples sandblasted by the Al_2_O_3_ powders under different pressures. Inset shows the strongest line ($11\bar{1}$) of monoclinic ZrO_2_ phase present in all the sandblasted samples.

To further investigate Y-TZP structural (and volume) changes discussed in this study, PXRD patterns of the ZR control and the sandblasted samples are given in Figure 3. PXRD is very applicable when studying diverse zirconia-based materials [12,28,29,50,51]. Figure 3 shows the presence of the tetragonal phase (but not the monoclinic phase), identified by the PCPDF2 card no. 01-088-1007, in the ZR sample. The above-mentioned tetragonal form of zirconia is responsible for the materials’ strength and toughness [52].

Furthermore, according to Figure 3, the sandblasting induced an expected (see, e.g., [28,29]) transformation of the tetragonal into the monoclinic phase, identified by the PCPDF2 card no. 00-005-0543. This transformation into the monoclinic phase, which can induce aging of the material’s surface [21,53], occured in all 16 sandblasted samples. On the other hand, according to this study [44], the Y-TZP surface treatment by laser does not yield monoclinic phase. However, according to the SEM study, the impact of the sandblasting was sufficient to induce severe surface damage. If both SEM and PXRD studies are taken into consideration, the sandblasting conditions should be selected depending on the induced surface’s damage. Moreover, Figure 3 demonstrates that the portion of the monoclinic phase in these samples is almost the same (ca. 10%). The volume increase (i.e., the monoclinic phase formation) confirms our hypothesis that it was responsible for sealing the holes that occurred in the ZR control sample due to CAM/CAD treatment (Figure 1).

### 3.4. Electrical Properties

**Figure 4:**
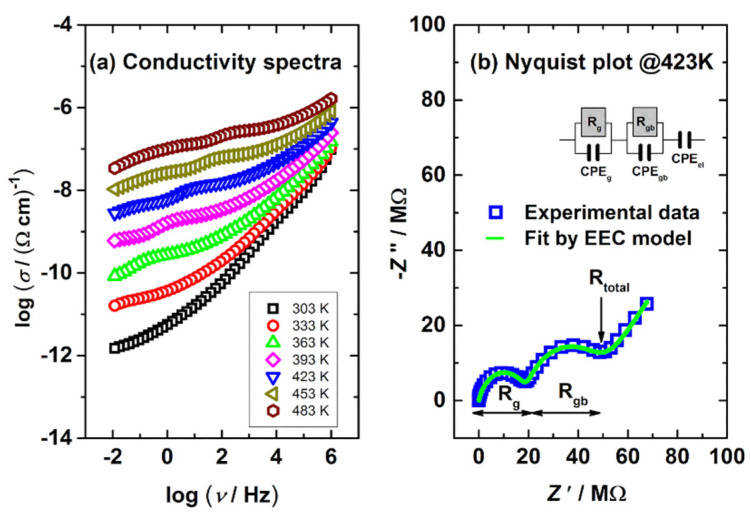
(**a**) Conductivity spectra and (**b**) Nyquist plot obtained at 423 K for the control sample ZR. EEC for the complex data fitting is shown in the (**b**) inset.

Zirconia-based materials (e.g., Y-TSZ) can be also considered as oxygen-ion conductive solid electrolytes [17,25]. The presence of oxygen vacancies in Y-TSZ allows the diffusion of O^2−^ ions within the material [54]. The Y-TZS conductivity depends on the dopant type and concentration [55], as well as on the phase arrangement [18,25]. Figure 4a,b show conductivity spectra and the Nyquist plot of the ZR sample obtained at various temperatures. The conductivity data (Figure 4a) were obtained in a wide range of frequencies (0.01–1 MHz). The ZR conductivity (Figure 4a) increases with the temperature, which is the expected behavior of ion-conductive material(s).

Next, complex Nyquist spectra (Figure 4b) obtained at 423 K display 2 arcs, a feature that corresponds well to the literature (e.g., [17]). The first arc in Figure 4b (characterized by R_g_) located in the high-frequency region can be attributed to intergrain (i.e., lattice) conductivity, while the second one (defined by R_gb_) can be assigned to intragrain (i.e., grain boundaries) [25]. Furthermore, the spur at low frequencies (a straight line in Figure 4a) is due to the surface-electrode effect. The proposed interpretation refers to ceramics, but it can be also used for any other solid materials with conductive crystalline grains, grain boundaries and similar conduction mechanism [56,57,58].

**Figure 5:**
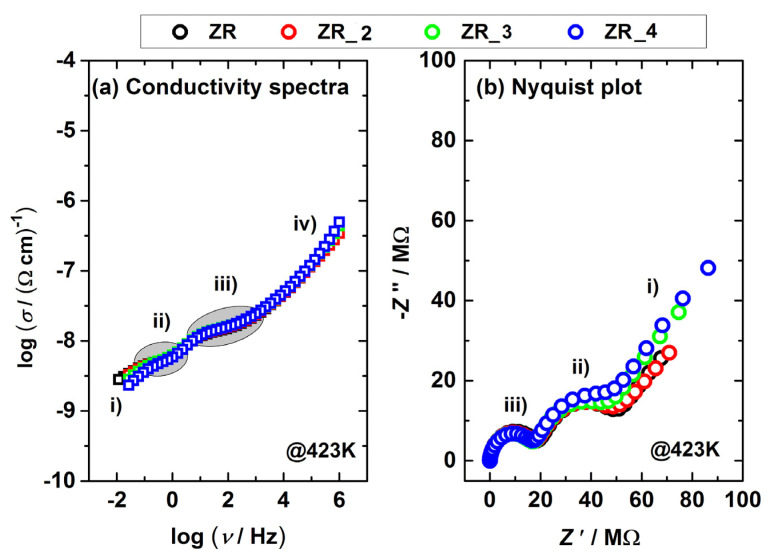
(**a**) Conductivity and (**b**) Nyquist spectra obtained at 423 K for selected samples (the control ZR and the sandblasted ZR_2, ZR_3, ZR_4). Different regions are marked as follows: (i) decrease/spur at lowest frequencies, (ii) low-frequency, (iii) middle-frequency region and (iv) dispersion (frequency-dependent region).

Furthermore, in order to investigate the impact of sandblasting, the conductivity and Nyquist plot of the ZR, ZR_2, ZR_3, ZR_4 samples recorded at 423 K are displayed in Figure 5a,b. According to Figure 5a, the conductivity of the samples in the low-frequency region is different, and this effect can also be detected in the second arc of Figure 5b. However, data fluctuation in the high-frequency region of Figure 5a,b is not so transparent; thus, an electrical equivalent circuit (EEC) model (inset in Figure 4b) was applied to fit the complex data displayed in Figure 5a.

EEC results (R_g_ and R_gb_) related to the samples’ resistance/conductivity (Figure 5a) are presented in Table 3, which show that a more intense sandblasting induced a decrease in both R_g_ and R_gb_ values. These specific phenomena can be attributed to both (a) the presence of the monoclinic phase and (b) the lower number of impurities in the sandblasted samples. To elaborate, the pure monoclinic phase is mainly an electronic conductor [17]; thus, the intergrain (i.e., lattice) resistance is lower and produces a declining R_g_ trend (Table 3). Even though the sandblasting induced various surface damages (Figure 2), this method also removes impurities from sample surfaces [15,59]. The lower number of impurities yields lower grain border resistance [25]; thus, the R_gb_ values shown in Table 3 show a downward trend. According to the authors’ knowledge and the available literature, this is the first time that electrical measurements (and EEC study) were used to monitor the impact of sandblasting on the number of impurities in dental material.

## 4. Conclusions

In this paper, sintered dental material of yttria stabilized-tetragonal zirconia polycrystals (Y-TZP) was studied. Differently prepared Y-TZP sample disks were prepared by CAD/CAM technology and were tested as-prepared and after the manual sandblasting by different sizes of Al_2_O_3_ powder under different amounts of pressure.

The SEM study showed holes of 5 µm in diameter in the ZR control sample disk, which can be assigned to CAD/CAM processing. However, such a sizable hole (5 µm in diameter) was not detected in the sandblasted samples. It was explained that the sandblasting induced a volume ingress that sealed such large holes. According to the surface investigations, it was found that the ZR_2 sample is most appropriate for dental application due to a less damaged surface.

Herein, the structural investigation pointed out that the monoclinic phase was not detected in the ZR control sample. After the sandblasting, XRPD patterns confirmed the existence of an additional monoclinic phase, which has 3–4% greater volume. The findings in this work clearly explain that transformation of the tetragonal into the monoclinic phase sealed holes that could appear after CAD/CAM treatment.

The conductivity measurements of ZR and the sandblasted sample disks reveal that the sandblasting (i) removed impurities from the sample surfaces which decreased the grain boundary resistance and (ii) induced transformation into the monoclinic phase that decreased intergrain (i.e., lattice) resistance.

Overall, the first highlight of this study is the fact that CAD/CAM treatment conducted in dental laboratories might induce holes (<5 µm) in the Y-TZP material. However, this work clearly demonstrated that these holes can be sealed by sandblasting due to transformation of the tetragonal into the monoclinic phase that is followed by the volume ingress. The second highlight is the application of both electrical measurements and EEC study to monitor a portion of impurities in Y-TZP material induced by sintering. It was shown that a lower number of impurities, obtained by sandblasting, decreased both grain boundary and lattice resistance.

## Figures and Tables

**Table 1:** Names of the samples obtained by sandblasting the ZR control sample disk with different Al_2_O_3_ particles and various amounts of pressure.

Size (Al_2_O_3_)/µm	Pressure/MPa			
	0.1	0.2	0.4	0.6
25	ZR_1	ZR_2	ZR_3	ZR_4
50	ZR_5	ZR_6	ZR_7	ZR_8
110	ZR_9	ZR_10	ZR_11	ZR_12
125	ZR_13	ZR_14	ZR_15	ZR_16

**Table 2:** Values of atomic fractions (%) of Zr and Al in the investigated sandblasted samples (see Table 1) obtained by EDX. ZR has 22.94% Zr and 0% Al.

	Samples							
	ZR_1	ZR_2	ZR_3	ZR_4	ZR_5	ZR_6	ZR_7	ZR_8
Zr/%	15.36	15.36	15.36	15.36	21.184	21.184	21.184	21.184
Al/%	10.87	10.87	10.87	10.87	9.024	9.024	9.024	9.024
	**Samples**							
	ZR_9	ZR_10	ZR_11	ZR_12	ZR_13	ZR_14	ZR_15	ZR_16
Zr/%	17.34	17.34	17.34	17.34	17.26	17.26	17.26	17.26
Al/%	10.31	10.31	10.31	10.31	9.83	9.83	9.83	9.83

**Table 3:** Selected EEC parameters obtained from fitting data given in Figure 5b.

EEC Parameters	Samples			
	ZR	ZR_2	ZR_3	ZR_4
R_g_/Ω	1.88 × 10^7^	1.72 × 10^7^	1.68 × 10^7^	1.63 × 10^7^
R_gb_/Ω	2.56 × 10^7^	2.42 × 10^7^	2.00 × 10^7^	1.69 × 10^7^

## Data Availability

Data sharing is not applicable to this article.
